# Prehospital measurement and treatment of ionised hypocalcaemia by UK helicopter emergency medical services in trauma patients: a survey of current practice

**DOI:** 10.1186/s13049-025-01379-2

**Published:** 2025-04-16

**Authors:** O. Hibberd, C. Leech, N. Lang, J. Price, EBG. Barnard

**Affiliations:** 1 Emergency and Urgent Care Research in Cambridge (EURECA) PACE Section, Department of Medicine, Cambridge University, Cambridge, UK; 2 Blizard Institute, Queen Mary University London, London, UK; 3 The Air Ambulance Service, Rugby, UK; 4 University Hospitals Coventry & Warwickshire NHS Trust, Coventry, UK; 5 Devon Air Ambulance, Exeter, UK; 6 Department of Research, Audit, Innovation, & Development (RAID), East Anglian Air Ambulance, Norwich, UK; 7 Academic Department of Military Emergency Medicine, Royal Centre for Defence Medicine (Research & Clinical Innovation), Birmingham, UK

**Keywords:** Calcium, Trauma, Haemorrhage, Transfusion, Prehospital, Helicopter emergency medical services

## Abstract

**Background:**

In the United Kingdom (UK), an increasing number of Helicopter Emergency Medical Services (HEMS) carry blood products for the resuscitation of patients with suspected haemorrhage. Ionised hypocalcaemia can occur due to calcium chelation from citrate-containing blood products or in response to traumatic injury. Therefore, many HEMS administer calcium alongside prehospital blood product transfusion. There are no national guidelines for prehospital calcium replacement. This study aimed to explore current UK HEMS protocols for calcium replacement associated with prehospital blood product transfusion and to report point-of-care testing (POCT) availability. The survey also sought to identify clinicians’ opinions on the measurement, significance, and management of trauma-induced ionised hypocalcaemia in the prehospital setting.

**Methods:**

A cross-sectional survey with single-staged purposive sampling was conducted between 26th September and 15th November 2024. The survey explored standard operating procedures (SOPs) for calcium replacement, the incidence of POCT, and clinicians’ opinions on the measurement and treatment of ionised hypocalcaemia. The survey was sent to the medical director, research lead, or a nominated clinician at the 21 HEMS in the UK on the 26th September 2024. These services were also invited to participate via a post on X (formerly Twitter) and a presentation delivered at the National HEMS Research and Audit Forum (NHRAF) on 26th September 2024.

**Results:**

21 HEMS responded to the survey (100% response rate), and all carried prehospital blood products and calcium replacement therapy. Eleven different combinations of blood products were carried. 20/21 (95%) had a SOP for calcium replacement during prehospital blood product transfusion. POCT of ionised calcium (iCa^2+^) was available at 6/21 (29%) of services. None had an SOP outlining the use of POCT for trauma patients, nor did any SOP specify the timing for measuring iCa^2+^. Clinicians’ opinions on the definition, measurement, and treatment of ionised hypocalcaemia varied widely.

**Conclusion:**

Blood products and calcium replacement therapy are now carried by all UK HEMS, but POCT is not in widespread use. Significant variation exists in the combination of products carried, protocols for calcium replacement, and opinions on the management of trauma-induced hypocalcaemia during prehospital transfusion, which suggests a need for further evidence.

**Supplementary Information:**

The online version contains supplementary material available at 10.1186/s13049-025-01379-2.

## Background

Major haemorrhage is one of the leading causes of preventable death in major trauma [1, 2]. It is widely acknowledged that the ‘lethal triad’ of coagulopathy, hypothermia, and acidosis can exacerbate haemorrhage [3]. Recently, ionised hypocalcaemia has also been identified as a contributing factor to this ‘lethal triad’, warranting its classification as the ‘lethal diamond’ [4, 5]. Ionised hypocalcaemia contributes to coagulopathy and cardiovascular decompensation due to its role in clot formation, vascular tone, and cardiac contractility [3, 5]. In the United Kingdom (UK), an increasing number of Helicopter Emergency Medical Services (HEMS) carry blood products for the resuscitation of patients with suspected haemorrhage [6–8]. Packed red blood cells (RBC), thawed fresh-frozen plasma (FFP), and dried plasma (DP, for example, lyophilised plasma) contain citrate as an anticoagulant, and blood product transfusion can lead to ionised hypocalcaemia due to calcium chelation with citrate [9–12]. In healthy individuals, the citrate in blood products can be quickly metabolised however, when haemorrhage and shock lead to hypoperfusion and hypothermia, citrate can accumulate, and the rapid infusion of citrate-containing blood products may exacerbate this accumulation [4, 13, 14]. Therefore, many HEMS administer calcium replacement alongside prehospital blood product transfusion [8]. Hypocalcaemia has also been reported prior to the administration of citrated blood products in trauma patients with severe haemorrhage, known as trauma-induced ionised hypocalcaemia [4, 14–16]. The mechanisms underlying this phenomenon are unclear and their interconnections not yet well defined, with a potentially complex and multifactorial aetiology involving haemodilution from crystalloid resuscitation, direct calcium loss from haemorrhage, intracellular calcium influx during ischaemia and reperfusion, and calcium-lactate binding [3–5, 17]. Trauma-induced ionised hypocalcaemia is associated with haemodynamic instability, increased transfusion requirements, and increased mortality [13–16, 18–26]. 

There are no national guidelines for prehospital calcium replacement and a significant variation in local protocols has previously been identified [8]. A 2022 survey reported that 6/25 (24%) critical care teams that carry blood products in the UK have the capability to measure ionised calcium (iCa^2+^) concentration using point-of-care testing (POCT). None of these services had a standard operating procedure (SOP) for measuring iCa^2+^ levels or thresholds for exogenous calcium replacement [8]. Since this survey, there has anecdotally been an increase in the availability of prehospital POCT equipment [27]. POCT iCa^2+^ measurement has the potential to identify early trauma-induced ionised hypocalcaemia. However, the incidence of this practice is unknown.

The aims of this study were to explore current UK HEMS protocols for calcium replacement associated with prehospital blood product transfusion and to report POCT availability. The survey also sought to identify clinicians’ opinions on the measurement, significance, and management of trauma-induced ionised hypocalcaemia in the prehospital setting.

## Methods

### Study design

A cross-sectional survey with single-staged purposive sampling was conducted between 26th September and 15th November 2024. The survey used Online Surveys V3 [28]. 

The 19-question survey (supplementary appendix) explored SOPs for calcium replacement, the incidence of prehospital POCT, and clinicians’ opinions on the measurement and treatment of trauma-induced ionised hypocalcaemia. Two HEMS representatives were used to validate the survey, and their returns were added to the final results.

Prehospital blood products were defined as either whole blood or by component therapy. Components included RBC, FFP, DP, and fibrinogen or prothrombin complex concentrate (PCC). Although platelet concentrate is considered as component therapy, this is not currently available to UK HEMS [29]. 

### Survey administration

**Fig. 1:**
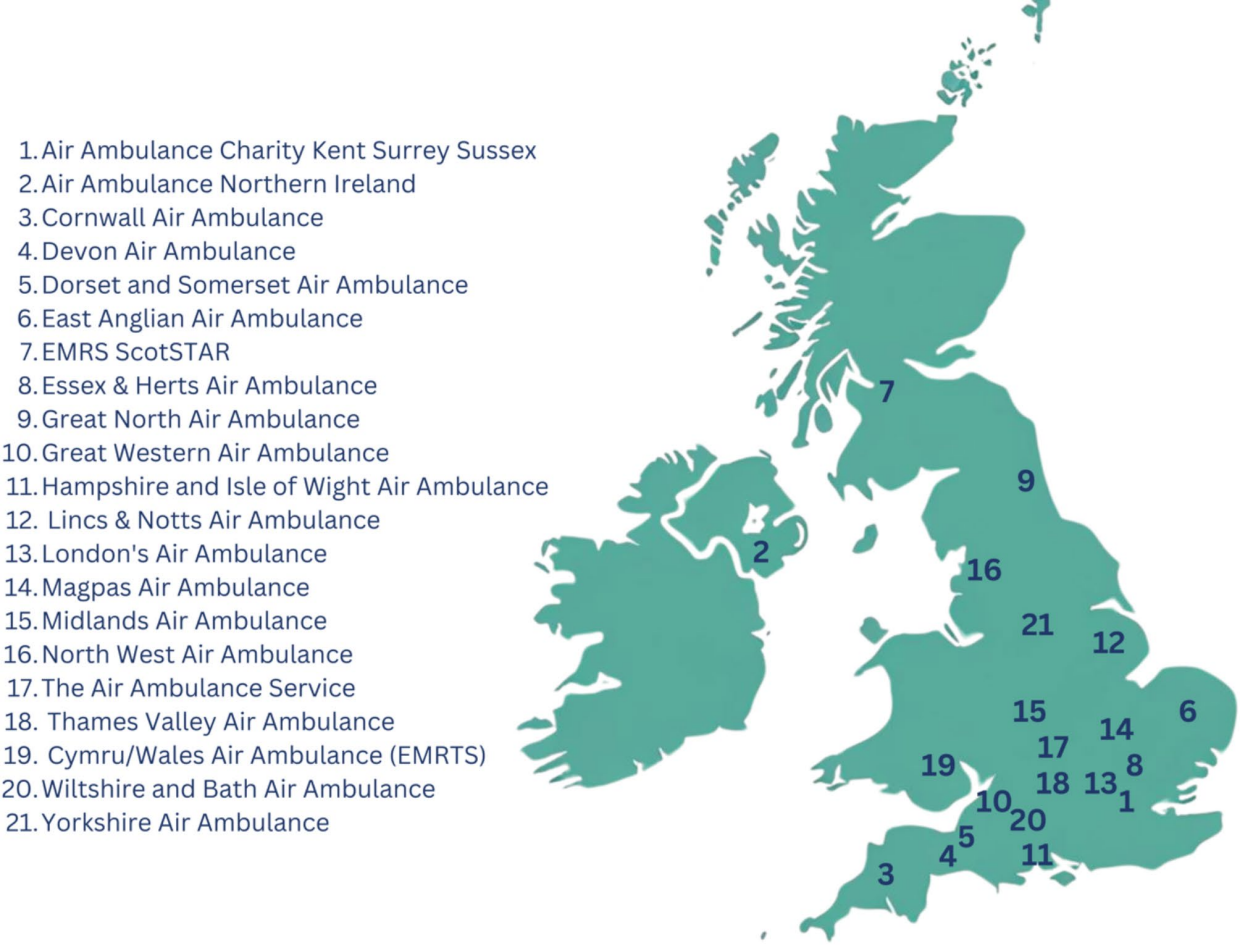
Approximate headquarters of all UK Helicopter Emergency Medical Services (*n* = 21)

The survey was sent by email to the medical director, research lead, or a nominated clinician at the 21 HEMS in the UK on the 26th September 2024 (Fig. 1).

These services were also invited to participate via a post on X (formerly Twitter) and a presentation delivered at the National HEMS Research and Audit Forum (NHRAF) on 26th September 2024. One response from each HEMS was recorded. A reminder email was sent to non-respondents after two weeks.

The study’s reporting followed the EQUATOR Network Consensus-Based Checklist for Reporting of Survey Studies (CROSS) checklist [30]. 

### Ethical considerations

No ethical approval was required as the survey results were collated anonymously, confidentially, and with no patient details included. The authors are the only people with access to the survey, which is General Data Protection Regulation (GDPR) compliant and certified to International Organisation for Standardisation (ISO) 27,001 standards. There was no patient or public involvement in this study’s design, conduct, or reporting.

### Statistical analysis

Results were reported descriptively as number (percentage). Missing data were also reported descriptively. No post-survey adjustments, such as imputation or weighting, were planned to address non-response bias. No sensitivity analysis was undertaken.

## Results

### Survey responses

21 HEMS responded to the survey (100% response rate). Duplicate survey responses were identified for one service; however, as the responses were identical, they were consolidated into a single entry. There were no missing data for responses related to prehospital blood products, calcium, and POCT.

21/21 (100%) of HEMS carried prehospital blood products. Amongst all services that carried prehospital blood products, 11 different combinations of blood products were reported (Table 1).

**Table 1:** Different combinations of blood products carried by UK HEMS (*n* = 21)

Blood products carried	Number of HEMS
Two units of RBC	2
Two units of RBC & Two units of FFP	5
Two units of RBC & Two units of DP	1
Two units of RBC, Two units of FFP, Two units of DP	1
Two units of RBC & Three units of DP	1
Three units of RBC	2
Four units of DP	1
Four units of RBC & Four units of FFP	5
Four units of RBC, Two units of FFP, Four units of DP	1
Four units of RBC, Four units of FFP, Four units of DP, Fibrinogen concentrate	1
Four units of RBC, Four units of FFP, 2500IU PCC	1

*RBC = Packed Red Blood Cells*

*FFP = Fresh Frozen Plasma*,* DP = Dried Plasma (e.g.*,* Lyophilised Plasma)*,* PCC = Prothrombin complex concentrate*

Packed red blood cells (RBC) were carried by 20/21 (95%), FFP by 14/21 (67%), and DP (lyophilised plasma) by 6/21 (29%) of services. Additionally, 1/21 (5%) carried Fibrinogen concentrate, and 1/21 (5%) carried PCC.

### Calcium replacement

21/21 (100%) of services carried calcium replacement therapy. 20/21 (95%) services administered a dose of 10mls of 10% calcium chloride, and 1/21 (5%) services administered 5mls of 10% calcium chloride. Four pre-filled 10 ml syringes were carried by 2/21 services (10%), three syringes by 6/21 (29%), two syringes by 9/21 (43%), and one syringe by 4/21 (19%) of services.

Among the 21 services carrying prehospital blood products, 20/21 (95%) had an SOP for calcium replacement during prehospital blood product transfusion. There were four distinct protocols regarding the timing of the initial calcium replacement. (Table 2).

**Table 2:** Different guidelines for calcium replacement during prehospital blood product transfusion amongst UK HEMS (*n* = 21)

Calcium replacement during prehospital blood product transfusion	Number of HEMS
No guidance on administration time	5
After (or just before) the first unit of blood product	5
After the second unit of blood product	7
During the fourth unit of blood product	1
After the fourth unit of blood product	3

### Point-of-care testing

POCT iCa^2+^ was available at 6/21 (29%) of services. Two services used the i-STAT^®^ 1 (Abbott Point of Care Inc., Princeton, USA) POCT device, one using both CG4 + and CG8 + cartridges and the other using CG8 + cartridges. Four services are using the EPOC^®^ Blood Analysis System (Siemens Healthineers, Erlangen, Germany). One service was undertaking feasibility testing with both the i-STAT^®^ with the CG8 + cartridge and the EPOC^®^ devices, and another service had purchased the EPOC^®^ device but had not yet introduced this into service. None of the services had an SOP outlining the use of POCT for trauma patients, nor did any SOP specify the timing for measuring iCa^2+^.

### Opinions on ionised hypocalcaemia management

15/21 (71%) and 19/21 (90%) responded to the hypothetical questions about calcium measurement and calcium replacement, respectively (supplementary appendix Table 1). Clinicians’ opinions on the definition of ionised hypocalcaemia varied widely among respondents, with definitions of mild/moderate/severe all overlapping (Supplementary Fig. 1). Definitions remained highly variable, irrespective of the availability of POCT for HEMS.

Hypothetical opinions were provided by 19/21 (90%) respondents regarding calcium administration for major trauma patients with iCa^2+^ who had not received a prehospital blood product transfusion. For patients with mild to moderate ionised hypocalcaemia prior to prehospital blood product transfusion, opinions varied on whether to administer a full dose (e.g. 10mls of 10% calcium chloride), half dose (e.g. 5mls of 10% calcium chloride), or no calcium. In cases of severe ionised hypocalcaemia, 12/19 (63%) respondents indicated they would administer a full dose of calcium, while the remainder preferred to withhold calcium until after prehospital blood product transfusion (Supplementary Fig. 2).

Quantified opinions on iCa^2+^ thresholds for initiating replacement in trauma patients who had not yet received prehospital blood product transfusions were provided by 11/21 respondents (52%). Approximately half of these respondents (5/11 (45%)) indicated they would replace calcium at levels below 1.0 mmol/L, while the remaining half (6/11(55%)) would initiate replacement at levels below 1.1 mmol/L.

### Future research

All UK HEMS responded that they would be interested to participate in further research exploring prehospital trauma-induced ionised hypocalcaemia.

## Discussion

Prehospital blood products are carried by all UK HEMS. All services with prehospital blood products also carry exogenous calcium replacement, with the majority carrying and administering a dose of 10mls of 10% calcium chloride. However, there are significant variations in the combinations of products carried and protocols for both prehospital blood product transfusion and calcium replacement. Approximately a quarter of services have no guidance on when to administer calcium during prehospital blood product transfusion. Similarly, around 25% of the services have the ability to undertake POCT of iCa^2+^. However, none of these services have established protocols specifying when testing should be undertaken for trauma patients. Hypothetically, if the iCa^2+^ was known to be low before prehospital blood product transfusion, over half of the clinicians would consider calcium replacement for the treatment of ionised hypocalcaemia but there was a lack of consensus on what level to start the replacement or what doses of calcium to provide.

This survey demonstrates an increased carriage of prehospital blood products in the UK. In 2018, ten HEMS were reported to carry blood products [31]. At a similar time, a survey of European HEMS across 14 countries observed that approximately half of the respondents carried blood products [32]. It is noteworthy that no UK HEMS carry whole blood, which is commonly utilised abroad, particularly in the United States [33]. There has also been an increase from a survey in 2022, which reported that 91% of HEMS carried blood products [8]. Despite the RePHILL study failing to demonstrate the superiority of RBC-Lyophilised Plasma (RBC-LyoPlas) to 0.9% sodium chloride for adult patients with trauma-related haemorrhagic shock [7], clinicians believe that prehospital blood product transfusion may be equitable to transfusion in the emergency department [8]. Results demonstrated point estimates toward a potential benefit regarding lactate clearance and increased survival at three hours for patients receiving prehospital blood products, but this did not reach significance [7]. However, the RePHILL trial was stopped early as a result of the COVID-19 pandemic, which prevented the study from recruiting its planned sample [7]. This survey also reports the variation in different blood product combinations, which demonstrates further challenges in generalising the results of the RePHILL study to all UK HEMS.

This survey also highlights significant variability in guidelines and practices related to calcium replacement therapy. The European guideline on the management of major bleeding and coagulopathy following trauma recommends that iCa^2+^ is maintained within the normal range and that 10mls of 10% calcium chloride is used to correct hypocalcaemia [1]. The guideline recommends calcium chloride over calcium gluconate because it contains a higher amount of calcium and is more suitable in cases of hypoperfusion and abnormal liver function, as patients receiving blood transfusions are less capable of clearing citrate [1]. However, the guideline does not specify when this should occur within treatment protocols without laboratory testing [1]. This study observed that approximately a quarter of protocols recommended replacing calcium after (or just before) the first prehospital blood product transfusion unit. This is likely due to the concern around trauma-induced hypocalcaemia and evidence that blood product transfusion can further reduce ionised calcium levels [11, 13, 16, 18, 19]. The administration of exogenous calcium can also act as an inotrope and a vasopressor, which may be advantageous for major patients with haemodynamic instability [34, 35]. 

However, arguments for exogenous calcium replacement at the point of injury and standardised protocolisation of replacement therapy is not as straightforward as it would initially seem. Studies exploring the use of calcium administration during cardiac arrest have not demonstrated any benefit, and a large randomised, placebo-controlled double-blind trial of calcium for out-of-hospital cardiac arrest was terminated early due to evidence of harm in the calcium administration arm [36, 37]. In trauma patients, hypercalcaemia has also been shown to be linked to mortality [18, 21, 38]. A propensity score-matched cohort of 28,323 adult major trauma patients, of whom 1,593 received supplemental calcium, demonstrated increased mortality among those who received calcium replacement (28.3% vs. 24.5%, *p* = 0.020) [21]. However, these results must be interpreted cautiously, as calcium was mainly administered to the most severely injured trauma patients [21]. When the results were adjusted for predicted mortality, the results were not significant (*p* = 0.244) [21]. Further data are needed to determine when to provide calcium replacement therapy for trauma patients before and during transfusion and to determine whether prehospital replacement risks detrimental hypercalcaemia. Overall, with the variety in protocols, opinions, and potential uncertainty about whether blind replacement may cause harm, the authors are unable to propose a recommendation based on this survey. However, the data presented here considerably enriches future research trajectories around what standards would represent safe and efficacious calcium replacement when treating the prehospital trauma patient.

POCT measurement of iCa^2+^ was available at six HEMS, with two further services planning to acquire equipment and remains unchanged from a previous survey on prehospital blood product transfusion [8]. POCT is commonly used to guide ventilation strategies and measure arterial carbon dioxide levels (PaCO_2_) in intubated patients with traumatic brain injury [39–41]. POCT may have particular utility in cases of prolonged evacuation or for rural HEMS [42, 43]. Despite the availability of testing, no service had a protocol that specified when to measure iCa^2+^ in trauma patients. POCT could allow the identification of ionised hypocalcaemia in the prehospital environment and guide replacement strategies. However, the current evidence base has not yet characterised how early trauma-induced ionised hypocalcaemia occurs in trauma patients and has not explored the context of ionised hypocalcaemia in the prehospital environment. Although testing has clear benefits, this must be balanced against the cost and practicalities of obtaining measurements in the prehospital environment and the displacement cost of other interventions. For example, there may not be time to complete testing in the most critically unwell patients for whom the provision of life-saving treatments takes priority but who are also the cohort most likely to benefit from iCa^2+^ measurement. Some studies have demonstrated the limitations of prehospital POCT [39, 44, 45]. Failure can result from sensitivity to temperature changes, vibration in a moving helicopter, and difficulty filling cartridges in the prehospital environment [39, 45]. Increased awareness of trauma-induced disturbances in ionised calcium levels would be a milestone whilst further research to determine the feasibility of the appropriate timing and efficacious use of testing POCT iCa^2+^ in trauma patients before prehospital blood product transfusion is needed.

### Limitations

There are several limitations of this survey. Not all providers of prehospital critical care (British Association for Immediate Care (BASICS) schemes or ambulance service critical care teams) that may carry prehospital blood products were included. Additionally, hypothetical questions related to the management of ionised hypocalcaemia are more likely to represent the personal views of respondents rather than a consensus opinion of each service; this may also have been the reason for the lower response rate related to these questions. However, having the survey completed by service clinical leads, leads for research, or nominated clinicians may have improved the generalisability of responses, whilst the results of these questions have utility for hypothesis generation. The protocols and opinions presented here are also not generalisable to a military setting where there is a higher proportion of penetrating and blast injury in which trauma-induced ionised hypocalcaemia is more likely [4, 46]. 

## Conclusion

Blood products and calcium replacement therapy are carried by all UK HEMS, but POCT is not in widespread use. Significant variation exists in the combination of products carried and the protocolised management of haemorrhage and calcium replacement during prehospital transfusion, which suggests a need for further evidence. Clinicians’ opinions on the measurement, significance, and management of trauma-induced ionised hypocalcaemia also varied widely.

## Electronic Supplementary Material

Below is the link to the electronic supplementary material.

**Supplementary Material 1**: **Supplementary Table 1**: Survey template for assessing UK HEMS measurement and supplementation of calcium in trauma.**Supplementary Figure 1**: Opinions on the definitions of ionised hypocalcaemia from respondents from UK helicopter emergency medicine services (*n* = 15).

**Supplementary Material 2**: **Supplementary Figure 2**: Hypothetical opinions on what doses of calcium would be preferred for the treatment of ionised hypocalcaemia in trauma patients who have not yet received prehospital blood product transfusion (*n* = 19).

## Data Availability

No datasets were generated or analysed during the current study.

## Abbreviations

BASICS: British association for immediate care
CROSS: Consensus-based checklist for reporting of survey studies
DP: Dried plasma
FFP: Fresh frozen plasma
GDPR: General data protection regulation
HEMS: Helicopter emergency medical services
iCa^2+^: Ionised calcium
ISO: International organisation for standardisation
PCC: Prothrombin complex concentrate
POCT: Point-of-care testing
RBC: Red blood cells
SOP: Standard operating procedure
UK: United Kingdom
