# Supplementary material for: Prehospital measurement and treatment of ionised hypocalcaemia by UK helicopter emergency medical services in trauma patients: a survey of current practice
Source: Scand J Trauma Resusc Emerg Med. 2025 Apr 16;33:63. doi: 10.1186/s13049-025-01379-2 (PMC12004557; doi:10.1186/s13049-025-01379-2)
Supplement: Supplementary file 1 — Supplementary Material 1: Supplementary Table 1: Survey template for assessing UK HEMS measurement and supplementation of calcium in trauma.Supplementary Figure 1: Opinions on the definitions of ionised hypocalcaemia from respondents from UK helicopter emergency medicine services (n = 15). [file 13049_2025_1379_MOESM1_ESM.docx]

**Supplementary appendix**

**Supplementary Table 1.** Survey Template for assessing UK HEMS measurement and supplementation of calcium in trauma

| **Survey Template** |
| --- |
| 1. **What organisation are you responding for?** Free text 2. **Does your service carry prehospital blood products?** Yes/No 3. **Which blood products do you carry and how many of these do you carry?** Free text 4. **Does your service carry calcium replacement therapy?** Yes/No 5. **If yes, please clarify the type and dose of calcium carried. For example, calcium chloride or gluconate, mls/%, duration of administration e.g. over ten minutes.** Free text 6. **How many syringes of calcium are carried?** Free text 7. **Does your service have an SOP for calcium replacement during blood transfusion?** Yes/No/We do not carry prehospital blood products 8. **If yes, what does the SOP advise regarding calcium replacement during prehospital blood product administration e.g. when should you routinely administer a bolus dose of calcium?** **Why does your service recommend this?** Free text 9. **Do you have any prehospital point-of-care testing for calcium?** Yes/No 10. **If yes, please specify the equipment used for point-of-care testing (POCT) of calcium (including the device and cartridge)**. Free text 11. **Do you have a SOP for when to use POCT for trauma patients?** Yes/No 12. **Does the SOP specifically include when to check calcium levels for trauma patients who are being considered or receiving prehospital blood products?** Yes/No/We don’t have an SOP for POCT/We don’t have POCT 13. **What levels of iCa (mmol/L) would you consider as mild?** Free text 14. **What levels of iCa (mmol/L) would you consider as moderate**? Free text 15. **What levels of iCa (mmol/L) would you consider as severe?** Free text 16. **At what level of iCa (mmol/L) would you consider administering supplemental calcium trauma patients who have not received blood?** Free text 17. **If administering pre-transfusion, would you opt for a full dose (10mmol 10%) or half dose (5mmol 10%)?** Full dose/half dose/we would not give calcium pre-transfusion 18. **Has your organisation been involved in previous or current prehospital calcium research and if so, what did this find?** Free text 19. **Would your organisation be interested to be involved in future research assessing prehospital calcium levels in trauma patients?** Yes/No |

Examples of nonquantifiable responses included “wide variation across service,” “no idea,” and “?”

**Supplementary Figure 1.** Opinions on the definitions of ionised hypocalcaemia from respondents from UK helicopter emergency medicine services (*n*=15)
