# Supplementary figures and images for: Prehospital measurement and treatment of ionised hypocalcaemia by UK helicopter emergency medical services in trauma patients: a survey of current practice
Source: Scand J Trauma Resusc Emerg Med. 2025 Apr 16;33:63. doi: 10.1186/s13049-025-01379-2 (PMC12004557; doi:10.1186/s13049-025-01379-2)

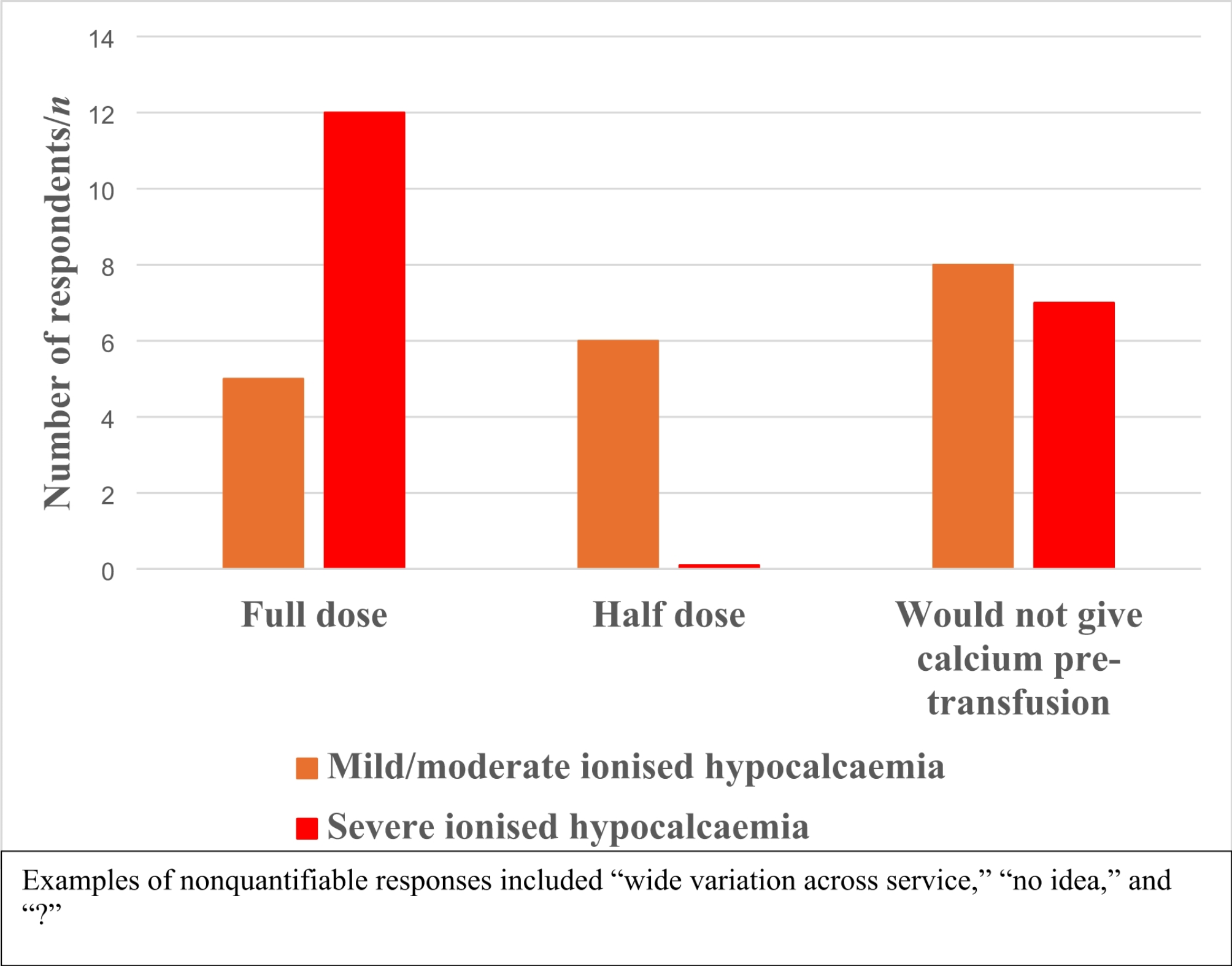

Supplement: Supplementary file 2 — Supplementary Material 2: Supplementary Figure 2: Hypothetical opinions on what doses of calcium would be preferred for the treatment of ionised hypocalcaemia in trauma patients who have not yet received prehospital blood product transfusion (n = 19). [file 13049_2025_1379_MOESM2_ESM.png]
